# Predictors of rate of change for children and youth with emotional disorders: a naturalistic observational study

**DOI:** 10.1186/s13034-016-0098-3

**Published:** 2016-05-05

**Authors:** Toril Sørheim Nilsen, Bjørn Helge Handegård, Martin Eisemann, Siv Kvernmo

**Affiliations:** Research Group For Clinical Psychology, Department of Psychology, Faculty of Health Sciences, UiT The Arctic University of Norway, 9037 Tromsø, Norway; Department of Child and Adolescent Psychiatry, Divisions of Child and Adolescent Health, University Hospital of North-Norway, P.O. Box 19, 9038 Tromsø, Norway; Regional Centre for Child and Youth Mental Health and Child Welfare, UiT The Arctic University of Norway, 9037 Tromsø, Norway; Research Group For Mental Ehealth, Department of psychology, UiT The Arctic University of Norway, 9037 Tromsø, Norway; Research Group of Pediatrics, Department of Clinical Medicine, Faculty of Health Sciences, UiT The Arctic University of Norway, 9037 Tromsø, Norway

**Keywords:** Predictors, Children, Emotional disorders, Anxiety, Depression, Treatment outcome, Outpatient

## Abstract

**Background:**

To examine demographic and clinical characteristics as potential predictors of change for children and youth with emotional disorders treated at two child and adolescent mental health outpatient services (CAMHS) in Norway.

**Methods:**

The study was of naturalistic observational type with “treatment as usual” (TAU). The sample consisted of 84 children and youth with emotional disorders. The Health of the Nation Outcome Scale (HONOSCA), and the Children’s Global Assessment Scale (CGAS) were administered at intake (T0), during the assessment (T1) and approximately six months after assessment (T2). Change was analysed by means of the linear mixed models procedure.

**Results:**

For the HONOSCA total score, youths with a diagnosis of depression had statistically higher symptom severity levels at baseline and significantly lower change rates as compared to youths with an anxiety disorder.

**Conclusions:**

The current study adds to the limited knowledge of predictors of rate of change for children and adolescents with emotional disorders treated within CAMHS. Our results point to a special need to improve clinical care for depressed children and adolescents. Important limitations comprising the external validity of the study concern missing data, a small study sample, and lack of information regarding the content and extent of the service provided.

**Electronic supplementary material:**

The online version of this article (doi:10.1186/s13034-016-0098-3) contains supplementary material, which is available to authorized users.

## Background

Depression and anxiety disorders are among the most prevalent problems presented in CAMHS in Norway [7, 35] and elsewhere [38, 41, 56]. Anxiety and depression often occur, both concurrently and sequentially [9, 14, 15]. The core emotions distinguish depressive disorder (depressed mood) and anxiety disorder (anxiety), while the secondary symptoms overlap considerably (e.g. difficulty with sleep, reduced concentration, rumination) [59]. Especially between depressive disorder and generalized anxiety disorders [31], and between social phobia and depressive disorders the overlap is considerable. Common biological markers as well as common risk factors have been described for these disorders. Research further suggests that different life events lead to the different disorders and that the prognosis for the two disorders differs [31]. Results from the Bergen child study (BCS) in Norway indicated that only 13 % of the group of children with anxiety or depressive disorder receive specialized mental health care [23].

For a better understanding of what kind of treatment is effective, it is of importance to identify and understand factors influencing treatment response [16, 34, 36]. Such knowledge may facilitate the process of targeting the treatment interventions to fit better individual client needs and to develop the most effective treatments. In the present study we examine potential predictors of rate of change [54, p. 137] during CAMHS interventions. Potential predictors of treatment change are numerous. Characteristics of the therapist (e.g. experience, theoretical orientation), family context (socioeconomic status, living situation, parental strain), parental context (marital satisfaction, psychopathology), and child characteristics (e.g. gender, age), are all potential predictors of treatment change.

Predictors of change have been primarily investigated in randomized controlled studies of cognitive-behavioural therapy in research clinics. To date, there is little consistent knowledge concerning predictors of change in anxiety and depression during and after psychiatric treatment in childhood. In summary, according to the majority of studies there is no association between demographic factors (gender and age) and change during treatment [11, 42]. Secondly, high baseline symptom severity and depression with comorbid anxiety are associated with a poorer treatment outcome in several studies of primary depression [10–12, 42]. Thirdly, associations between general or non-internalizing comorbidity and depression treatment outcome have most often been non-significant [42, 45]. Fourthly, the majority of findings from the anxiety studies suggests that severity and comorbidity do not impact on change during treatment [42, 45], but several studies also indicate that higher severity of anxiety and comorbid internalizing (i.e. other anxiety or depressive) disorder are predictive of less change during treatment [3, 17, 37, 44, 55]. A few studies have indicated that social functioning and conflicts may impact on change during treatment in depression [20, 28, 51] and in anxiety disorders [51].

### Aims and hypothesis

There is a need for more knowledge regarding factors influencing change during treatment in outpatient CAMHS. The aim of this study was to examine demographic and clinical characteristics of patients as potential predictors of rate of change in the clinician-rated HONOSCA (total score) and the CGAS, during child and adolescent psychiatric outpatient treatment in a naturalistic sample of patients with anxiety and/or depressive disorders (hereafter referred to as emotional disorders). In the current study, change during treatment was conceptualized as the average rate of change per month on symptomatic level and functional impairment scores. Throughout the text, we use the term “rate of change” to refer to the observed differences in symptomatic- and functional impairment level during the period of service provision. When referring to other treatment outcome studies we use the general term “change” to conceptualize any approach to the definition of change during treatment. The following research question was addressed:

1. Predictors of rate of change over time:
  - Does any of the pre-treatment demographic or clinical characteristics predict rate of change over time in HoNOSCA and CGAS? The characteristics tested were age, gender, baseline symptom severity or functional impairment, type of emotional disorder, comorbidity, prosocial characteristics and problem with peers. We also examined whether there were differences between the two clinics in HoNOSCA and CGAS rates of change.

## Methods

### Subjects and setting

The present study is part of a larger multicenter study including seven child and adolescent mental health clinics (CAMHS) in the Northern and the South-eastern parts of Norway. The multi-center study was a naturalistic observational study where data from clinical instruments were collected as part of ordinary clinical practice. Since treatment practice was not changed as a result of the ongoing observational study, the treatment given can be classified as “treatment as usual” (TAU). The content, type and the extent of the treatment provided were not recorded in this study. Clinicians’ verbal accounts in retrospect of what constituted “treatment as usual” indicate that no particular therapeutic or theoretical approach took precedence at the clinics, but where chosen according to the individual clinicians’ competence. Both cognitive-behavioural- and psychodynamic approaches were used and both individual and family-based interventions were offered. For depression and anxiety disorders, medical treatment was not first line treatment, but was in a few cases offered as additional treatment.

Among the 320 clients eligible for this part of the multi-centres study, only 276 patients had data for two or more measurement occasions. In the present study, a subsample of 84 patients with emotional disorders treated at two CAMHS in the north of Norway was the target group. The two centres, CAMHS Alta (n = 56) and CAMHS Silsand (n = 28), were the only clinics within the multi-centre study collecting follow-up data. The two centres had similar population composite with both a rural and semirural population base. Based on accounts from clinical staff, there are no obvious overall differences in the type of treatment offered at the two centres. Characteristics of the CAMHS Alta, the CAMHS Silsand sample, and the multicentre sample are presented in Additional file 1: Table S1, whereas characteristics of the study sample are presented in Table 1. The study sample consists of 56 girls (66.7 %) and 28 boys (33.3 %). The mean age of the sample was 12.49 years at intake, and the girls (*M* = 13.21, *SD* = 2.65) were significantly older (t (82) = −3.24, *p* < .01) than the boys (*M* = 11.04, *SD* = 3.38). Twenty-seven patients (32.2 %) were assessed as depressed (4 boys and 23 girls), 38 patients (45.2 %) as having one or more anxiety disorders (18 boys and 20 girls), and 19 patients (22.6 %) were assessed as having both depressive and anxiety problems (6 boys and 13 girls). The children and adolescents included in this study will in the following be referred to as “children”. A follow-up (T2) assessment was not completed by the clinicians for 32.1 % (n = 27) of the sample for the HONOSCA and 38.1 % (n = 32) for the CGAS. The reason for non-completion is unknown. The group of patients without follow-up data was not different from the rest of the sample as regards gender composition, mean age, age grouping and type of emotional disorder (depression, anxiety or mixed). Test statistics for comparison of the groups are presented in Additional file 1: Tables S2, S3.

**Table 1:** Descriptives for the sample

	Measures	
	CGAS	HONOSCA
N	82	80
Gender % (n)		
Male	32.9 (27)	33.8 (27)
Female	67.1 (55)	66.3 (53)
Age (Mean/SD)	12.6 (2.99)	12.51 (2.98)
Age group % (n)		
0–6 years	2.4 (2)	2.5 (2)
7–12 years	40.2 (33)	41.3 (33)
13–18 years	57.3 (47)	56.3 (45)
Emotional disorders % (n)		
Anxiety	46.3 (38)	46.3 (37)
Depression	32.9 (27)	32.5 (26)
Mixed anx/depr	20.7 (17)	21.3 (17)
Duration of problems % (n)		
Less than a month	–	–
1–5 months	8.8 (7)	8.5 (7)
6–12 months	10.0 (8)	9.8 (8)
More than 12 months	42.5 (34)	42.7 (35)
Missing	38.8 (31)	39.0 (32)
Family arrangement		
Both parents	30.5 (25)	30 (24)
Part time mum/dad	7.3 (5)	7.5 (6)
Either mum/dad	34.1 (28)	35 (28)
Parent/stepparent	8.5 (7)	8.8 (7)
Grandpar/relatives	–	1.3 (1)
Fosterhome	3.7 (3)	3.8 (3)
Institution	4.9 (4)	3.8 (3)
Alone	1 (1.2)	1.3 (1)
Other	1 (1.2)	1.3 (1)
Ethnicity (mother)		
Norwegian	90.2 (74)	90 (72)
European	1.2 (1)	1.3 (1)
Missing	8.5 (7)	8.8 (7)
Ethnicity (father)		
Norwegian	85.4 (70)	85 (68)
European	1.2 (1)	1.3 (1)
Sami	1.2 (1)	1.3 (1)
African	1.2 (1)	1.3 (1)
Missing	11 (9)	11.3 (9)
Repeated measurement		
All assessments	46.3 (38)	61.3 (49)
T0–T1	20.7 (17)	17.5 (14)
T1–T2	6.1 (5)	7.5 (6)
T0–T2	8.5 (7)	3.8 (3)
One assessment only	18.4 (15)	9.9 (8)

### Procedure

**Fig. 1:**
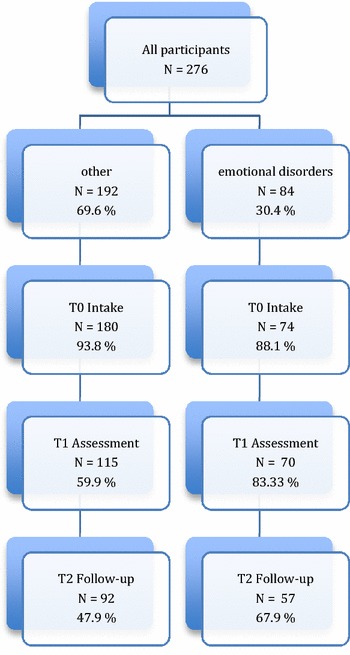
Flowchart of participants: Clinician rated HoNOSCA

**Fig. 2:**
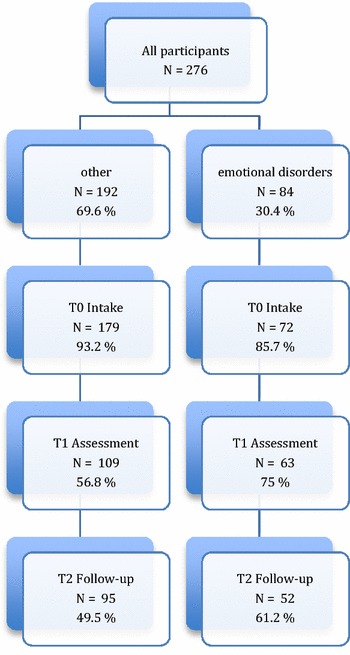
Flowchart of participants: Clinician rated CGAS

In this multi-centre study, all children and youths between the ages of 5 and 18 referred to the clinics between 2002 and 2005 were asked to participate. The only exclusion criteria were acute referral and age <5 years. Refusal to participate in the study did not affect the service offered, and non-participants were assessed and treated by the same procedures as study participants. Measures by means of questionnaires were repeated on three occasions. The clinician rated HONOSCA and the CGAS were administered at intake (T0), during assessment/treatment (T1) and approximately 6 months after the assessment (T2). At T1, The Kiddie-SADS PL, a semi-structured diagnostic interview (age range 6–18 years), was used to aid diagnostic evaluation [1, 30], and diagnosis. Due to the problem of incomplete data, we had to rely on different selection procedures to identify the greatest number of relevant cases. First, we used the Kiddie-SADS interview to identify children and youth that fulfilled the criteria for a diagnosis of unipolar depression and/or a diagnosis of one or several anxiety disorder (n = 57). Two raters, Toril Sørheim Nilsen and Siv Kvernmo, rated all the interviews independently. Bjørn Helge Handegård, calculated the inter-rater agreement. The Gwet’s AC2 per disorder is presented in Additional file 1: Table S4. Furthermore, cases with disparate ratings were discussed and consensus based diagnoses were set. Finally, cases with missing data for the Kiddie-SADS, but with a registered axis 1 diagnosis of depression and/or anxiety, were selected (n = 27). In the clinics, diagnoses were consensus based and evaluated by a specialist in clinical psychology or psychiatry (Figs. 1, 2).

The study had been approved by the Regional Committee for Medical and Health Research Ethics of Northern Norway.

### Measures

#### Diagnostic interview

The Schedule for Affective Disorders and Schizophrenia for School-Age Children-Present and Lifetime version (K-SADS-PL) was used as the diagnostic interview during assessment [1, 30]. The interview provides DSM-IV diagnoses of a wide range of psychiatric disorders. First, the clinician conducted the screening interview with a primary caregiver and the child/adolescent. Problem areas being discovered during the screening were further assessed through targeted supplements. For our sample the most common supplements were: 1. Affective disorders (n = 29), and/or supplement 3. Anxiety disorders (n = 34). Other supplements conducted in this sample were: Supplement 4. Behavioural disorders (n = 18), Supplement 5. Drug abuse and other disorders (n = 5), and Supplement 2. Psychotic disorders (n = 2). The interviews were all conducted by clinicians trained and experienced with the Kiddie SADS. No further interrater reliability tests were done in the clinics.

#### The Health of the Nation Outcome Scale (HONOSCA)

The HONOSCA is a 15-item clinician-rated measure of mental health symptoms in children and adolescents. The HONOSCA items are scored on a 5-point scale from 0 (no problem) to 4 (severe to very severe problems) with a maximum total score of 52. The Norwegian version of HoNOSCA has been found to have good psychometric properties, with good inter-rater reliability, good sensitivity to change, and good concurrent and criterion-related validity [49]. The HONOSCA have also been found to be sensitive to change in clinical populations [4, 5, 18, 19, 24, 32, 33, 47]. In the present study, total score changes were evaluated. Cronbach’s α for the HONOSCA total score at intake was .52 for the current sample. The HONOSCA total score has proven as a good quantitative measure of clinical severity [5, 19] and correlates well with the CGAS [38, 60].

#### The Children’s Global Assessment Scale (CGAS)

The CGAS [52] is a clinician-rated score in the range from 0 (needs constant supervision) to 100 (superior functioning in all areas). The CGAS is being extensively used as a measure of change in global functioning. The psychometric properties of the CGAS show moderate reliability and validity [38, 39, 48, 53]. The CGAS has been used as one “gold standard” for psychosocial functioning when validating other instruments [50]. Several recent large-scale outcome studies, both naturalistic studies and randomized controlled trials, have included change in the CGAS as one outcome measure [38, 60]. The Norwegian version of the CGAS is currently being evaluated for its psychometric properties.

### Predictor variables

Potential predictors were demographic and clinical characteristics recorded at baseline or during the assessment. *Clinic* was coded as 0 (CAMHS Alta) and 1 (CAMHS Silsand). *Gender* was coded as 0 (male) and 1 (female). *Age* at intake was centred, and the mean age for the sample of patients with emotional disorders was 12.49 years (SD = 3.07, min–max 4–18). *Baseline severity:* The HONOSCA total score at baseline was tested as a continuous predictor of change over time in the CGAS. *Baseline functioning:* Baseline CGAS—scores was tested as a continuous predictor of change in the HONOSCA total score. *Comorbidity* as a covariate was assessed by comorbid disorders through the Kiddie-SADS interview dichotomous variable (0 no comorbid disorder, 1 one or more comorbid disorders). The strenghts and difficulties questionnaire (SDQ) *prosocial* scale (self- and mother reported) was used to assess social competence, and was coded as a continuous variable with a scale from 0 through 10. The SDQ *peer problem* scale (self- and mother reported) was coded as a continuous variable with a scale from 0 through 10.

### Statistical analysis

All statistical analyses were performed using SPSS version 22.0. Longitudinal multilevel analysis, also known as the mixed models approach, was used in this study. When evaluating the effects of predictors of rate change and of baseline symptom severity and functional impairment we assessed the random intercept and the random slope to see whether individual variances in initial status or rate of change were statistically significant, and thus whether there were variability that could be explained by potential predictors. Potential predictors were tested individually as covariates in the fixed effects part of the model. We evaluated the interaction effect between the variables with time onto the dependent variables.

### Multilevel-model-based fit indices and total variability explained

The likelihood ratio test [46] was used to assess the improvement in fit from the random intercept model to the random intercept and random slope model. Singer and Willett [52–54] [pp. 102–103] account of the pseudo-*R*^2^ statistic was used. We calculated the pseudo-*R*^2^ statistic of the total outcome variability that was explained by the predictors in the model, and we assessed change in the pseudo-*R*^2^ statistic when adding a predictor. According to Singer and Willett [54] this pseudo-*R*^2^ must be interpreted with caution, since total outcome variation is partitioned into several variance components.

## Results

Results of the mixed models analysis with the HONOSCA and the CGAS as dependent variables are presented in Tables 2 and 3, respectively. Explanations of the tables’ parameters in relation to the different predictor variables are presented in the supplemental material. The results regarding the average rate of change for the HONOSCA and the CGAS (the fixed effects of time) has been presented in prior work [43] and we only present the main-findings for the clinician-reported measures here. First, the average change rates per month (fixed slopes) indicate statistically significant improvement in total severity (HONOSCA: β_01_ = −.52, SE = 0.06, p < .001) and in psychosocial functioning (CGAS: β_01_ = .98, SE = .17, p < .001). Looking at the effect sizes, the pseudo-*R*^2^ showed small to moderate associations between predicted and observed scores with 18 % of change in the HONOSCA total score and 12 % of the change in the CGAS being associated with linear time. For the clinician rated measures, the change rate during the active assessment/treatment period (T1–T2) seem to be larger than the average change rates of the waitlist periode (T0–T1). Finally, from the perspective of clinically significant change, only a small proportion of subjects could be classified as recovered and/or improved. For the majority of subjects, the degree of change was uncertain.

### Random effects

Results for the random effects part of the model are not presented in the Tables, but will be shortly presented here. Residual variation on level 1 was highly significant for all models, indicating the potential to include time-varying predictors. The random intercept was also significant in all models, with a few exceptions. The random slope and the intercept-slope covariance were not statistically significant in any models, with a few exceptions.

### Clinic

Results of the mixed models analysis with *clinic* as a covariate in the model showed that for the HONOSCA total score there were no significant differences in total severity at baseline or in rate of change over time between the CAMHS Alta and the CAMHS Silsand samples. Results for the CGAS showed statistically significant differences between the clinics in baseline predicted mean scores (CAMHS Alta: *β*_01_ = 66.78; CAMHS Silsand: *β*_01_ = 57.76; t = 3.44, p < .01) and in rate of change per month (CAMHS Alta: *β*_11_ = .72, SE = .44; CAMHS Silsand: *β*_11_ = 1.73, t = −2,31; p < .05).

### Predictors of change over time in the HONOSCA and CGAS

The random slopes were not statistically significant for any model, which implies little between-patient variability in the development over time in the HONOSCA and CGAS. The likelihood ratio test showed a statistically significant improvement in fit of the model with the CGAS as change measure (χ^2^ (2) = 35.81, *p* < .01), but not for the HONOSCA total score: (χ^2^ (2) = 1.57, *p* = *ns*). Despite this, we chose to explore potential predictors of rate of change in the HONOSCA, as well.

Results of the mixed models analysis with the HONOSCA as the dependent variable are presented in Table 2. Individuals with a diagnosis of depression had lower rates of change than individuals with a diagnosis of anxiety (β_01_ = *−*.29, SE = .13, *p* < .05). Also, individuals with a diagnosis of depression had significantly higher baseline scores when compared to individuals with anxiety disorders (β_01_ = 2.63, SE = 1.29, *p* < .05). The pseudo-*R*^2^ statistics of total variability explained, ranged from 15 % (the model with baseline CGAS as predictors) to 26 % (diagnosis: depression vs mixed) in the model with the HONOSCA total score as the dependent variable. The pseudo-*R*^2^ statistics increased from 18 % (model with no predictor) to 26 % in the model with diagnosis (depression vs mixed) as a predictor.

Results of the mixed models analysis with the CGAS as the dependent variable are presented in Table 3. None of the tested variables were significant predictors of change over time in the CGAS. The model with no predictor explained 12 % of the total variability. The pseudo-*R*^2^ statistics of total variability explained with the CGAS score as the dependent variable, ranged from 10 % (baseline HoNOSCA) to 30 % (self-reported prosocial characteristics).

**Table 2:** Longitudinal analysis of the HONOSCA total score with demographic and clinical factors as covariates, with Pseudo-*R* ^2^ (total variability explained)

	Fixed effects				Pseudo-*R* ^2^
Variable	Intercept	Time (month)	Predictor	Time X predictor	
Predictor variable					
No predictor	13.50 (.58)***	−.52 (.06)***	–	–	.18
Gender	13.43 (.71)***	−.58 (.08)***	0.22 (1.24)	0.16 (.13)	.19
Age	13.35* (.58)***	−.52 (.06)***	0.27 (.20)	−.01 (.02)	.19
CGAS (baseline)	16.26 (4.90)**	−1.46 (.72)*	−0.05 (.07)	.01 (.01)	.15
Depression vs anxiety	12.24 (.83)***	−.40 (.08)***	2.63 (1.29)*	−.29 (.13)*	.20
Depression vs mixed	14.12 (1.15)***	−.57 (.14)***	0.74 (1.48)	−.12 (.20)	.26
Anxiety vs mixed	14.16 (1.29)***	−.57 (.13)***	−1.92 (1.55)	.17 (.15)	.15
Comorbidity	12.48 (.81)***	−.56 (.09)***	1.88 (1.05)	.08 (.11)	.25
SDQ scores at baseline					
Prosocial scale (mother-report)	16.90 (2.44)***	−.39 (.30)	−.54 (.30)	−.01 (.04)	.19
Peerproblems (mother-report)	10.68 (.87)***	−.47 (.10)***	.71 (.26)**	.00003 (.03)	.24

* *p* < .05, ** *p* < .01, *** *p* < .001 table parameters in Table 2 through 9 are explained in Additional file 1

**Table 3:** Longitudinal analysis of the CGAS score with demographic and clinical factors as covariates, with Pseudo-R^2^ (total variability explained)

Variable	Fixed effects				Pseudo*-R* ^2^
	Intercept	Time (month)	Predictor	Time X predictor	
Predictor variable					
No predictor	64.03 (1.32)***	.98 (.17)***	–	–	.12
Gender	65.21 (1.46)***	1.04 (.20)***	−0.3.78 (2.56)	−0.16 (.35)	.16
Age	64.12 (1.35)***	.95 (.17)***	−0.05 (.45)	−0.06 (.06)	.12
HoNOSCA (baseline)	70.29 (5.14)***	.24 (.91)	−0.33 (.38)	0.06 (.07)	.10
Depression vs anxiety	64.58 (1.98)***	.86 (.24)**	−1.16 (3.06)	0.35 (.41)	.14
Depression vs mixed	63.48 (2.74)***	.96 (.32)**	0.001 (3.51)	0.15 (.32)	.16
Anxiety vs mixed	63.92 (2.85)***	.89 (.40)*	0.66 (3.46)	−0.03 (.48)	.10
Comorbidity	67.14 (1.88)***	.84 (.24)**	−4.94 (2.47)*	0.06 (.31)	.15
SDQ scores at baseline					
Prosocial scale (mother-report)	56.71 (5.42)***	.90 (.83)	1.11 (.67)	−0.003 (.10)	.14
Peerproblems (mother-report)	68.18 (1.96)***	.82 (.28)**	−0.97 (.58)	0.02 (.08)	.13

* *p* < .05, ** *p* < .01, *** *p* < .001

## Discussion

To sum up the main findings of this study: firstly, there were statistically significant differences between the clinics in the ratings of functional impairment at baseline and in the rate of change per month. Children in the CAMHS Silsand sample had significantly higher CGAS scores at baseline and a significantly higher rate of change as compared to the CAMHS Alta sample. Secondly, children with a diagnosis of depression had statistically higher symptom severity levels at baseline, and significantly lower rates of change in symptom level as compared to children with an anxiety disorder. The remaining variables were not statistically significant predictors of rate of change in clinician-reported total severity. Among the variables tested here, none were significant predictors of rate of change in functional impairment. The main findings listed above will be further discussed.

The patient group at CAMHS Silsand had lower initial CGAS, and a higher CGAS rate of change than the CAMHS Alta patient group. On the other hand the clinics did not differ as regards to the corresponding HONOSCA scores. The finding may reflect actual differences between the two clinics in the impact of problems for their respective patient groups, and also a difference in the rate of change for the samples of their patients. The finding may also reflect what is known as regression toward the mean (RTM), the tendency for high intitial scores to follow a reductionist path and to be closer to the mean at follow-up [29]. Differences between the clinics may also reflect local differences at the two clinics in how the CGAS scale is implemented and scored rather than an actual difference between baseline levels and rate of change of the patient groups in the two clinics. The interrater reliability of the HoNOSCA (ICC = .84) has been found to be significantly higher than the CGAS (ICC = .61) in a large international study [22].

Children with a diagnosis of depression were rated by clinicians as having higher levels of symptoms at baseline and as experiencing less change, when compared to children with an anxiety diagnosis. This finding is in line with research showing that anxiety disordered youths are more likely than depressive youths to recover if treated. A meta-analysis of cognitive-behavioural therapy (CBT) for youth depression show remission rates of 48 % for CBT and 34 % to placebo [57], while remission rates of CBT for youths with an anxiety disorder were 57 % for CBT and 35 % for placebo [8, 27], respectively. Another meta-analysis of psychotherapy for anxiety disorders based on 24 randomized controlled trials (all CBT treatment) found a recovery rate of 68.9 %, and an effect size of .82 [26]. A review of the current treatment of pediatric depression (both psychotherapy and pharmacotherapy) estimated remission rates of depression to be 60 % within 6 months [40]. A meta-analysis of the selective re-uptake inhibitor (SSRI) fluoxetine [6], showed a response rate of 61 % for depressed youth (50 % response to placebo) and 69 % response rate for anxiety disordered youth (39 % response to placebo).

Clinician-rated functional impairment at baseline and rate of change, were not different between depressed as compared to youths with an anxiety disorder. Thus, depressed and anxiety disordered children were assessed as equally impaired with regards to psychosocial functioning at baseline. In a large-scale study of psychiatric treatment outcomes in CAMHS in Stockholm, Sweden, both baseline and change scores of the CGAS for depressive and anxiety-disordered youth were comparable with our finding [38].

None of the demographic pre-treatment variables emerged as significant predictors of rate of change in clinician-rated measures. Thus, gender and age did not impact on the rate of change. One plausible interpretation of this finding may be that the outpatient service provided, functioned equally well for both genders and for different age groups within this sample of patients. The apparent lack of association between demographic factors, such as age and gender, and change during treatment, has been a consistent finding in a meta-analysis of depression [58] and in literature reviews and studies addressing predictors of change in treatment studies of depression or anxiety disorders [2, 11, 13, 25, 42]. Thus, one of the most consistent findings regarding predictors of change is that age and gender do not seem to impact on change rates or rates of remission.

### Limitations

This study has several limitations. Firstly, as in many naturalistic observational studies, the problem of missing data could have influenced our results. By using the Mixed Models approach, some of the problems with missing data were accounted for since this method allows for the inclusion of subjects with missing data. Missing data includes both missing information for variables tested as predictors, and also the reduction of the number of respondents from T0 through T1 to T2. This may raise questions about the representativeness of the results.

Secondly, a further limitation was the low number of clients in this study, and statistical analyses performed on small samples may partly explain the lack of effects in our study. The multi-centre CAMHS North study was not originally designed to examine predictors and moderators of change. In the project plan 300–500 clients were expected to be included in the study, and thus the study was originally powered to examine mechanisms of change. As in many naturalistic observation studies the problem of missing data for repeated measurement was considerable. Among the 320 clients eligible for this part of the multi-centre study, only 276 patients had data for two or more measurement occasions. Among the 276, only 190 had available data for the diagnostic interview Kiddie-SADS. Since the research questions targeted interaction effects, and the testing of predictors of rate of change, our sample of 84 patients may have been too small to detect small and intermediate effects. Correlations between predictors and outcomes are often of small to moderate magnitude, and thus large samples are needed to achieve sufficient power (n > 200 if r = .2 and power = .8 in correlational analyses) [21]. Also, some of the clinical characteristics tested as potential predictors should preferably have been tested with more refined categories (e.g. type of comorbid disorder), but due to few clients in most subcategories we decided to dichotomize these variables.

A third limitation, which may compromise the external validity of the results of this study, is the lack of some relevant information about the service provided. Such information could be about type of interventions, number of sessions, clinician’s competence and overall caseload, and reasons for dropouts. This compromises the opportunity to correct for potentially important characteristics with the service that could have impacted on the results. Further, in this study we included potential predictor variables that were available within this multicentre study. Many potential predictors of change were not available for assessment in this study.

On the other hand, one advantage of the present study is that it was carried out in a naturalistic setting without exclusion criteria, except for age <5 years and acute referral. We could only find few studies which report findings regarding predictors of change in CAMHS outpatient settings. Another advantage was the evaluation of predictors of rate of change over time in both symptom severity and functional impairment. In addition, we assessed the impact of multiple potential predictors, both demographic and clinical.

### Implications and recommendations

The results of the current study have implications for both clinical practice and research in clinical settings. Routinely collecting data about the rate of change in patients is an important first step in order to identify for whom the treatment works or not. Our results are in line with other clinical studies that imply the need to improve clinical care and treatment, especially for depressed children and adolescent. It is important to note that the apparently worse prognosis for depressed patients as compared with anxiety disordered patients, both in our study and other clinical studies, may be partly due to different mechanisms within the two conditions other than the services provided. The centres may benefit from a good implementation plan including training and continued monitoring for mental health professionals.

Regarding implications for research in clinical settings, we have several recommendations for similar future studies in naturalistic settings. The issue of missing data and of accomplishing complete datasets for clinical and follow-up data is a recurrent issue in service research [see e.g. 2, pp. 45–46]. We became aware of the importance of having administrative resources to monitor the data collection process. In this multicentre study, the one clinic (CAMHS Alta) with such a resource also had more complete data. The lack of information about both the extent and content of the service provided in the current study, limits the generalizability of results and also the clinical implications of the information that can be gained from this particular service setting. Thus, we will stress the importance of having staff monitoring the ongoing collection of service data to ensure the registration of important service information.

## Conclusions

The current study adds to the limited knowledge of predictors of rate of change and predictors of baseline symptom severity and functional impairment for children and adolescents with emotional disorders treated within CAMHS. Naturally, the results need to be replicated in future studies. There is a great need of well-planned, and carefully monitored studies in naturalistic settings to address the research questions raised here. The results presented here point to a special need to improve clinical care for depressed children and adolescents.

## Additional file

10.1186/s13034-016-0098-3 Characteristics of the CAMHS Alta and the CAMHS Silsand sample. **Table S2**. Comparison of groups with/without T2 data. **Table S3**. Comparison of groups with/without T2 data. **Table S4**. Inter-rater reliability based on Gwet’s AC2 for Kiddie-SADS diagnoses (current episode). **Table S5**. Explanation of table parameters.

## Abbreviations

ADHD: attention-deficit hyperactivity disorder
CAMHS: child and adolescent mental health outpatient services
CBT: cognitive-behavioral therapy
SSRI: selective re-uptake inhibitor
ADAPT: the adolescent depression antidepressant and psychotherapy trial
BCS: the Bergen child study
CAMS: the child/adolescent anxiety multimodal treatment study
CGAS: The Children’s Global Assessment Scale
SDQF: the father-reported SDQ
HoNOSCA: the health of the nation outcome scale
SDQM: the mother-reported SDQ
SDQS: the self-reported SDQ
K-SADS-PL: the schedule for affective disorders and schizophrenia for school-age children-present and lifetime version
SDQ: the strengths and difficulties questionnaire
TAU: treatment as usual
TADS: treatment of adolescents with depression study
TORDIA: treatment of resistant depression in adolescents study
