# Supplementary material for: Predictors of rate of change for children and youth with emotional disorders: a naturalistic observational study
Source: Child Adolesc Psychiatry Ment Health. 2016 May 5;10:11. doi: 10.1186/s13034-016-0098-3 (PMC4857241; doi:10.1186/s13034-016-0098-3)
Supplement: Supplementary file 1 — 10.1186/s13034-016-0098-3 Characteristics of the CAMHS Alta and the CAMHS Silsand sample. Table S2. Comparison of groups with/without T2 data. Table S3. Comparison of groups with/without T2 data. Table S4. Inter-rater reliability based on Gwet’s AC2 for Kiddie-SADS diagnoses (current episode). Table S5. Explanation of table parameters. [file 13034_2016_98_MOESM1_ESM.docx]

Table S1. Characteristics of the CAMHS Alta and the CAMHS Silsand sample

|  | CAMHS Alta | CAMHS Silsand | Total sample |
| --- | --- | --- | --- |
|  |  |  |  |
| N | 56 | 26 | 236 |
| Gender % (n) |  |  |  |
| Male | 26.8 (15) | 46.2 (12) | 58.1 (137) |
| Female | 73.2 (41) | 53.8 (14) | 41.9 (99) |
| Age (Mean/SD) | 12.16 (2.9) | 13.54 (2.9) | 10.48 (3.6) |
| Age group % (n) |  |  |  |
| 0-6 years | 1.8 (1) | 3.8 (1) | 14.4 (34) |
| 7-12 years | 46.4 (26) | 28.9 (7) | 53.0 (125) |
| 13-18 years | 51.8 (29) | 69.2 (18) | 32.6 (77) |
| Emotional disorders % (n) | |  |  |
| Anxiety | 48.2 (27) | 42.3 (11) | - |
| Depression | 28.6 (16) | 42.3 (11) | - |
| Mixed anx/depr | 23.2 (13) | 15.4 (4) | - |
| Family arrangement % (n) |  |  |  |
| Both parents | 28,6 (16) | 35.7 (10) | 37.7 (89) |
| Part time mum/dad | 7,1 (4) | 7.1 (2) | 6.4 (15) |
| Either mum/dad | 37,5 (21) | 25 (7) | 28.4 (67) |
| Parent/stepparent | 12,5 (7) | - | 12.3 (29) |
| Grandpar/relatives | 1,8 (1) | - | 0.4 (1) |
| Fosterhome | 3,6 (2) | 7.1 (2) | 6.8 (16) |
| Institution | 0 | 14.3 (4) | 1.7 (4) |
| Alone | 1,8 (1) | - | - |
| Other | 0 | 3.6 (1) | 0.4 (1) |
| Ethnicity (mother) % (n) |  |  |  |
| Norwegian | 87.5 (49) | 96.2 (25) | 88.1 (208) |
| European | 1.8 (1) | - | 0.4 (1) |
| Sami | - | - | 1.3 (3) |
| Nordic | - | - | 0.8 (2) |
| African | - | - | 0.8 (2) |
| Asian | - | - | 0.4 (1) |
| Latin-American | - | - | 0.4 (1) |
| Ethnicity (father) % (n) |  |  |  |
| Norwegian | 83.9 (47) | 88.5 (23) | 82.6 (195) |
| European | 1.8 (1) | - | 0.4 (1) |
| Sami | 1.8 (1) | - | 1.3 (3) |
| African | - | 3.8 (1) | 1.7 (4) |
| Nordic |  |  | 0.4 (1) |
| Asian |  |  | 0.4 (1) |
| Mother tongue |  |  |  |
| Norwegian | 85.7 (48) | 96.2 (25) | 86 (203) |
| Bilingual | 3.6 (2) | - | 1.7 (4) |
| Other | - | - | 0.4 (1) |
| Repeated measurement |  |  |  |
| HONOSCA |  |  |  |
| All assessments | 78.6 (44) | 17.9 (5) |  |
| T0-T1 | 10.7 (6) | 32.14 (9) |  |
| T1-T2 | 3.6 (2) | 10.7 (3) |  |
| T0-T2 | 5.4 (3) | 0 |  |
| One assessment only | 1.8 (1) | 25 (7) |  |
| No assessment | - | 14.3 (4) |  |
| CGAS |  |  |  |
| All assessments | 60.7 (34) | 17.9 (5) |  |
| T0-T1 | 14.3 (8) | 10.7 (3) |  |
| T1-T2 | 1.8 (1) | 7.5 (6) |  |
| T0-T2 | 12.5 (7) | 0 |  |
| One assessment only | 10.7 (6) | 32.14 (9) |  |
| No assessment | - | 7.14 (2) |  |

Table S2: Comparison of groups with/without T2 data

| Characteristic | t | Mean difference | St error difference | sig |
| --- | --- | --- | --- | --- |
| Age | .44 | .32 | .72 | ns |

Table S3: Comparison of groups with/without T2 data

| Characteristic | χ | df | sig |
| --- | --- | --- | --- |
| Age group | 3.15 | 2 | ns |
| Gender composition | 0 | 1 | ns |
| Diagnosis group | 1.08 | 2 | ns |

Table S4: Inter-rater reliability based on Gwet´s AC2 for Kiddie-SADS diagnoses (current episode)

| Diagnoses | Gwet´s AC2 | StdError |
| --- | --- | --- |
| Major depressive disorder | 0.90 | 0.03 |
| Dysthymia | 0.95 | 0.02 |
| Depressive disorder NOS | 0.96 | 0.01 |
| Adj. disorder w depressed mood | 0.94 | 0.02 |
| Panic disorder | 0.99 | 0.01 |
| Separation anxiety disorder | 0.94 | 0.02 |
| Simple phobia | 0.97 | 0.01 |
| Social phobia | 0.95 | 0.01 |
| Agoraphobia | 0.98 | 0.01 |
| Generalized anxiety disorder | 0.92 | 0.02 |
| Obsessive-compulsive disorder | 0.98 | 0.01 |
| Post-traumatic stress disorder | 0.96 | 0.02 |
| Adj. disorder w anxious mood | 0.92 | 0.02 |

Table S5: Explanation of Table parameters

|  |  | *Fixed effects* | | | |
| --- | --- | --- | --- | --- | --- |
| Variable | | **Intercept** | **Time (month)** | **Predictor** | **Time X predictor** |
| Gender | | Baseline HONOSCA/CGAS/SDQ for girls | Average change rate per month in HONOSCA /CGAS/SDQ for girls | Gender difference in baseline HONOSCA/CGAS/SDQ | Gender difference in rate of change in HONOSCA /CGAS. |
| Age | | Baseline scores for mean aged children (12.43 years) | Rate of change for mean aged children | Difference in HONOSCA/CGAS/ SDQ baseline score by one year increase in age | Difference in rate of change in HONOSCA /CGAS with one year increase in age |
| Severity  CGAS/HoNOSCA (baseline) | | Baseline HONOSCA for children with baseline CGAS = 0 | Time = Rate og change for children with CGAS = 0 | Difference in HONOSCA baseline score by one point increase in the CGAS baseline score | Difference in rate of change in HONOSCA with one point increase in baseline CGAS |
| Depression vs Anxiety | | Average HONOSCA/ CGAS/SDQ baseline score of children with anxiety disorder | Average change rate per month of children with anxiety disorders; | Difference in baseline HONOSCA/CGAS/SDQ for children with depression vs children with anxiety disorders | Difference in rate of change in HONOSCA/CGAS between children with depression vs anxiety disorders. |
| Depression vs Mixed | | Average HONOSCA/ CGAS/SDQ baseline score of children with mixed anxiety and depressive disorders | Average change rate per month in HONOSCA/CGAS/SDQ of children with mixed disorders | Difference in baseline HONOSCA/CGAS/SDQ for children with depression vs children with mixed disorders | Difference in rate of change in HONOSCA/CGAS between children with depression vs mixed disorders. |
| Anxiety vs Mixed | | Average HONOSCA/CGAS/SDQ baseline score of children with mixed disorders | Average change rate per month in HONOSCA/CGAS/SDQ of children with mixed disorders | Difference in baseline HONOSCA/CGAS/SDQ for children with anxiety vs children with mixed disorders | Difference in rate of change in HONOSCA/CGAS between children with anxiety vs mixed disorders. |
| Comorbidity | | Average HONOSCA/CGAS/SDQ at baseline for individuals with one or several comorbid disorders | Average rate of change per month in HONOSCA/CGAS/SDQ for individuals with one or several comorbid disorders; | Difference in baseline HONOSCA/CGAS/SDQ between individuals with one or more comorbid disorders and individuals with no comorbid disorder | Difference in rate of change in HONOSCA/CGAS between individuals with one or more comorbid disorders and individuals with no comorbid disorder |
| SDQ scores at baseline | |  |  |  |  |
| SDQ peerproblem/ prosocial score | | Average HONOSCA/CGAS at baseline for individuals with SDQ score = 0 | Average rate of change per month in HONOSCA/CGAS for individuals with SDQ score = 0. | Change in baseline HONOSCA/CGAS by one point change in SDQ score | Difference in rate of change in HONOSCA/CGAS by one point change in SDQ score |
